# Miscellany

**Published:** 1888-08

**Authors:** 


### Miscellany.

THE following circular relating to the approaching Congress ot American Physicians and Surgeons is published for the information of those interested:

THE CONGRESS OF AMERICAN PHYSICIANS AND SURGEONS.

WASHINGTON, D. C., July, 1888.

Circular No. 3.

The Committee of Arrangements takes pleasure in announcing to the members and invited guests of the special societies taking part in the Congress, that the arrangements are sufficiently advanced to assure the success of the first triennial session of the Congress of American Physicians and Surgeons, which will be held in the City of Washingion during the eighteenth, nineteenth and twentieth of September next.

A number of distinguished physicians and surgeons have signified their acceptance of the invitation to attend, among whom may be named Sir Spencer Wells, Sir Andrew Clark, Sir William McCormac, Drs. W. O. Priestley, William Ord, and Grainger Stewart, Mr. Lawson Tait, Mr. Victor Horsley, Mr. Thomas Bryant, Mr. Thomas Annandale, Professors Ferrier, Esmarch and Gerhardt, Drs. Rafael Lavista, of Mexico; J. L. Reverdin, of Geneva; O. W. Holmes and H. J. Bowditch, of Boston; Joseph Leidy, of Philadelphia; W. Kingston and Eccles, of Canada.

The preliminary programmes of the participating societies have been published. It is hoped the classified and final programmes will be forwarded without further delay. Several are already in possession of the committee. As soon as all are received, the final programme of the Congress will be printed and distributed.

Places of meeting for the Congress and each of the societies have been secured, conveniently located, so that members may interchange attendance without annoyance.

The meetings of the Congress will be held during the evenings, beginning at eight o'clock P. M.; on the evenings of the eighteenth and nineteenth, the meetings will be held in the main hall of the Grand Army Building, 1412 and 1414 Pennsylvania avenue, and on the last (Thursday evening) in the hall of the National Museum. During the evening the Army Medical Museum and Library Building, along side of the Museum building, will be lighted and opened for the inspection of the members and invited guests. The meetings of the societies will be held during the day, according to the programme each may respectively provide. The sessions will be open to the profession.

On Monday evening, September 17th, a dinner will be given by the members of the Congress to the guests of the participating societies. Invitations to this dinner will be sent only to the specially invited guests who have indicated their acceptance. The contributing members will receive cards of admission. It will be limited exclusively to members of the Congress and invited guests.

An informal collation will be served at Willard's Hotel on Tuesday evening, after the adjournment of the meeting of the Congress, to the guests and those members who may choose to attend. A similar entertainment will be served in the National Museum Building on Thursday night, after the final adjournment of the Congress.

Guests are requested to notify the Chairman immediately after their arrival in Washington, giving their address and stating whether they have ladies with them. Special arrangements will be made for the entertainment of the wives and daughters of the guests.

Hotel accommodations are ample and conveniently located to the places of meeting.

The Secretaries of the special societies are requested to forward to the Chairman the names and addresses of their foreign guests.

Members of the Congress and the guests are expected to register. A parlor in Willard's Hotel will be provided for that purpose, from which the mail of the members and guests will be distributed, and at which the city residence of each member or guest can be ascertained.

All communications should be addressed to the Chairman of the Committee.

SAMUEL C. BUSEY, M. D.,
1545 I street north-west, Washington City,
*Chairman Committee of Arrangements.*

---

### Places of Meeting of the Congress and Medical Societies
### *In Washington, during Sept. 18, 19 and 20, 1888.*

The Congress of American Physicians and Surgeons: Tuesday and Wednesday nights, main hall, Grand Army Building, 1412 and 1414 Pennsylvania avenue; Thursday night, hall in National Museum, Smithsonian Grounds.

American Surgical Association: Main hall, Grand Army Building, 1412 and 1414 Pennsylvania avenue.

Association of American Physicians: Hall No. 1, Grand Army Building, 1412 and 1414 Pennsylvania avenue.

American Climatological Association: Hall No. 2, Grand Army Building, 1412 and 1414 Pennsylvania avenue.

American Laryngological Association: Hall No. 3, Grand Army Building, 1412 and 1414 Pennsylvania avenue.

American Dermatological Association: Parlor in Willard's Hotel.

Association of Genito-Urinary Surgeons: Parlor in Willard's Hotel.

American Neurological Association: Parlor in Willard's Hotel.

American Orthopedic Association: Welcker's Hotel, Fifteenth street, North Washington.

American Otological Society: Arlington Hotel, Vermont avenue.

American Physiological Society: Army Medical Museum.

American Ophthalmological Society: Arlington Hotel, Vermont avenue and H street.

American Gynecological Society: Columbian University, Fifteenth and H streets, North Washington.

American Association of Obstetricians and Gynecologists: National Medical College Building, H street, North Washington.

Office of Registration, Willard's Hotel.

The above announcement is made for the information of the medical profession.

SAMUEL C. BUSEY, M. D.,

*Chairman Committee of Arrangements.*

---

THUDICHUM has made a special study of the alkaloids found in human urine. They are six in number: Urochrome, urotheobromine, creatinine, reducine, parareducine and aromine. Urochrome is the principal coloring matter of the urine; its origin is not known, as its products of decomposition show no relation to the coloring matters of the blood and bile. Aromine is the odorous constituent of urine. Urotheobromine is isomeric with the alkaloid of cacao.

---

JABCONSKI, of Poitiers, favors the isolation of tubercular patients, and proposes an ordinance excluding consumptive children from the public schools.

---

THERE is now in the Museum of Natural History, in Paris, a remarkable living monstrosity nine months of age. It has one brain, two trunks united, four arms and four legs. Teratologists classify it as a *monencephalic thoradelphus*.

